# Possible association of the *Plasmodium falciparum* T1526C *resa2* gene mutation with severe malaria

**DOI:** 10.1186/1475-2875-11-128

**Published:** 2012-04-25

**Authors:** Rémy Durand, Florence Migot-Nabias, Valérie Andriantsoanirina, Elise Seringe, Firmine Viwami, Gratien Sagbo, Francis Lalya, Philippe Deloron, Odile Mercereau-Puijalon, Serge Bonnefoy

**Affiliations:** 1 Laboratoire de Parasitologie-Mycologie, Hôpital Avicenne, AP-HP, 125 rue de Stalingrad, 93009, Bobigny Cedex, France; 2 UMR 216 Mère et enfant face aux infections tropicales, Paris, France; 3 Institut de Recherche pour le Développement, Paris, France; 4 Faculté de Pharmacie, Université Paris Descartes, Sorbonne Paris Cité, France; 5 Hôpital Pitié Salpêtrière, AP-HP, Université Pierre et Marie Curie, Paris, France; 6 Centre d’Etude et de Recherche sur le Paludisme Associé à la Grossesse et l’Enfance (CERPAGE), Cotonou, Benin; 7 Service de Pédiatrie, Centre National Hospitalier et Universitaire Hubert K. Maga, Cotonou, Benin; 8 Institut Pasteur, Unité d’Immunologie Moléculaire des Parasites, CNRS URA 2581, Paris, France

**Keywords:** *Plasmodium falciparum*, Severe malaria, Ring-infected erythrocyte surface antigen

## Abstract

**Background:**

*Plasmodium falciparum* exports proteins that remodel the erythrocyte membrane. One such protein, called Pf155/RESA (RESA1) contributes to parasite fitness, optimizing parasite survival during febrile episodes. *Resa1* gene is a member of a small family comprising three highly related genes. Preliminary evidence led to a search for clues indicating the involvement of RESA2 protein in the pathophysiology of malaria. In the present study, cDNA sequence of *resa2* gene was obtained from two different strains. The proportion of *P. falciparum* isolates having a non-stop T1526C mutation in *resa2* gene was evaluated and the association of this genotype with severity of malaria was investigated.

**Methods:**

*Resa2* cDNAs of two different strains (a patient isolate and K1 culture adapted strain) was obtained by RT-PCR and DNA sequencing was performed to confirm its gene structure. The proportion of isolates having a T1526C mutation was evaluated using a PCR-RFLP methodology on groups of severe malaria and uncomplicated patients recruited in 1991–1994 in Senegal and in 2009 in Benin.

**Results:**

A unique ORF with an internal translation stop was found in the patient isolate (Genbank access number : JN183870), while the K1 strain harboured the T1526C mutation (Genbank access number : JN183869) which affects the internal stop codon and restores a full length coding sequence. About 14% of isolates obtained from Senegal and Benin harboured mutant T1526C parasites. Some isolates had both wild and mutant *resa* alleles. The analysis excluding those mixed isolates showed that the *resa2* T1526C mutation was found more frequently in severe malaria cases than in uncomplicated cases (p = 0.008). The association of the presence of the mutant allele and parasitaemia >4% was shown in multivariate analysis (p = 0.03) in the group of Beninese children.

**Conclusions:**

All T1526C mutant parasites theoretically have the ability to give rise to a full-length RESA2 protein. This study raises the hypothesis that the RESA2 protein could favour high-density infections. Other studies in various geographic settings and probably including more patients are now required to replicate these results and to answer the questions raised by these results.

## Background

The reasons why certain *Plasmodium falciparum*-infected patients develop severe malaria (SM) complications are unclear. Most studies on SM have focussed on cyto-adhesion of erythrocytes infected with late stage parasites, immunological disorders or host genetic susceptibility [1]. However, SM is usually associated with a huge parasitic load, leading to excessive local parasite burden. The parasite, which reproduces by a 10^12^ factor in a few weeks, has evolved pathogenicity and virulence factors that determine the infection outcome. Variations in virulence may result from an increased parasite capacity to uptake nutriments and/or a more efficient efflux of waste material, high multiplication rate, optimal remodelling of the host infected erythrocytes and resistance to host defenses such as fever and adaptive immune response. In this regard, ring stages have received little attention, despite their critical role in parasite installation into its host cell and their exposure to host responses. Ring stages are exposed to fever, a hallmark of malaria disease, which is triggered by toxic materials released upon rupture of the infected erythrocyte. Febrile temperatures are detrimental to parasite development of late [2] but also of young stages [3,4].

As *P. falciparum* enters the erythrocyte, it progressively modifies the structural components and the machinery of its host cell in order to create an adequate environment and to overcome host responses. Thus, immediately after invasion, *P. falciparum* exports proteins that remodel the erythrocyte membrane, modifying its mechanical, functional and antigenic properties. One such protein, called Pf155/RESA (RESA1), localized in the erythrocyte membrane upon invasion and interacting with the erythrocyte cytoskeleton protein spectrin, stabilizes the infected red blood cell cytoskeleton, thereby allowing it to overcome membrane weakening upon exposure at febrile temperatures [4-6]. Thus, ring stage expression of RESA1 contributes to parasite fitness, optimizing parasite survival during febrile episodes. RESA1 has also been shown to be targeted by the adaptive immune response in populations living in endemic areas. Antibodies reacting with RESA1 inhibited erythrocyte invasion [7-10] moreover were associated with protection against clinical malaria [11-15]. Completion of the *P. falciparum* genome sequence showed that *resa1* gene is a member of a small family comprising three highly related genes (PFA0110W *resa1*, PF11_0512 *resa2* and PF11_0509 *resa3*) that share similar erythrocytic stage transcription profile. The role of RESA3 is still unknown.

The *resa2* gene (accession number M91672) was first reported as a pseudogene based on the presence of an internal stop codon [16]. Interestingly, SNP affecting the third base of this internal stop codon is described in PlasmodB with the replacement of the internal stop codon by a Glutamine codon in some laboratory parasite strains (e.g. K1). This T1526C mutation may restore a unique open reading frame and thus the ability to give rise to a full-length protein. Large differences in *resa2* gene transcription *in vitro* have been reported [16,17] and increased transcription of this gene was observed after parasite exposure at 41°C [3]. *In vivoresa2* behave as a transcribed pseudogene [18] and analysis of gene expression profiles in malaria patients has consistently outlined *resa2* expression and up-regulation *in vivo*[18-20]. In addition, a preliminary study indicated that isolates with high parasitaemia harboured frequently mutated T1526C *resa2* gene (data not shown). Thus, preliminary evidence led to a search for clues indicating the involvement of RESA2 in the pathophysiology of malaria. In the first part of the present study, the *resa2* cDNA gene structure from two different strains with or without the T1526C mutation was determined. RFLP study was performed to investigate the presence of the T1526C mutation in *P. falciparum* isolates obtained in two geographical settings in Benin and Senegal and the association of this genotype with the severity of malaria attack was investigated.

## Methods

### Study areas and sample collection

Senegalese patients and samples have been described in Robert *et al*[21]. Briefly, two groups of patients were recruited at the Hôpital Principal de Dakar. The first group corresponded to SM patients (aged <1 to 63 years) admitted to the Intensive Care Unit (27 children and 29 adults). All presented at least one of the symptoms defined by the World Health Organization [22] as criteria for severe malaria (severe anaemia, altered conciousness (Glascow Coma Scale score of ≤ 9), convulsions, hypoglycaemia, acidosis, respiratory distress and impaired visceral functions). The second group consisted of 30 outpatients with an uncomplicated malaria attack (UM) (see Additional file  1 and Additional file  2). From April to August 2009, 101 children with symptomatic *P. falciparum* malaria were recruited in the CNHU of Cotonou, Benin. These children lived in the urban area of Cotonou where malaria transmission is perennial, with two seasonal peaks corresponding to rainy seasons, from April to July and September to November. A survey conducted in 2000 showed heterogeneous malaria transmission in the city of Cotonou, with transmission varying from five, 29 and 47 infective bites per person per year near the beach, in the centre of the city and in the outer-urban lagoon areas, respectively [23].

The inoculation rates were undoubtedly lower during the study period, due to the introduction of effective prevention and therapy. At admission, children were assigned to the SM or the UM group as described elsewhere [24]. Children age, gender, place of residence, anti-malarial drug intake by the patient, and duration of symptoms prior to enrolment were documented by questionnaire. For each individual a 5 mL venous blood was collected in EDTA Vacutainer® tubes before drug treatment administration. Peripheral parasitaemia at admission was assessed by microscopy on Giemsa-stained blood films and blood smears. In addition, for each individual, two drops of fresh blood were collected onto filter paper for molecular study.

Characteristics of the recruited Beninese subjects are presented in Additional file  3 and Additional file  4. Forty-seven subjects and 54 subjects were included in the UM group and in the SM group, respectively. The sex ratio (M/F) of the SM group [1.7 (34/20)] did not differ significantly from that of the UM group [1.35 (27/20)]. Subjects with UM were older than children with SM (mean; SD 107.2 months; 156.6 *vs* 34.8 months; 21) (Mann Withney test, p < 0.001, for comparison between the groups). Patients of the SM group had higher parasite density than patient of the UM group (mean density 10.53% *vs* 3.57%). Forty-seven children had severe malarial anaemia only, one child had cerebral malaria only, and six children had both severe anaemia and cerebral malaria.

This Beninese study was approved by the ethics committee of the “Faculté des Sciences de la Santé” of the University of Abomey-Calavi in Benin. For each child, a written informed consent from parents or legal guardians was obtained. The study was conducted in accordance with the Declaration of Helsinki.

### *In vitro* culture growth assay

Leukocytes were removed from infected blood following dilution with equal volume of phosphate-buffered saline (PBS) and separation on Ficoll-Paque density gradient. Depleted blood was washed three times in PBS. Infected erythrocytes were suspended in complete RPMI culture media supplemented with 10% human AB-positive serum at a haematocrit of 5%. For isolates with parasitemia >5%, dilution with washed non-infected 0+ RBC obtained from a unique non-*P. falciparum* infected donor was performed. Parasites were cultured for 72 hours at 37°C using the candle jar method [25] and culture medium was changed daily.

#### DNA extraction

Parasite DNA was extracted from blood spots with Instagene® Matrix resin (BioRad℗, Marnes la Coquette, France), according to the manufacturer’s instructions.

#### DNA genotyping

Oligonucleotides used for the polymerase chain reaction (PCR) were selected in order to specifically amplify the RESA2 sequence and to not cross-hybridize with RESA1 or RESA3. PCR amplifications were performed in 50μL reaction buffer (50 mM KCl, 10 mM Tris–HCl, pH8.3) containing 3 μL of DNA solution, 0.4 μM each primer 5′TGATGCCGTAAAAGATGGTG3′ (sense) and 5′TCATATCTGCATTTATATCGACACCT′ (antisense), 250 μM each dNTP, 3 mM MgCl_2_, and 1.25 U of *Thermus aquaticus* DNA polymerase (AmpliTaq Gold; PerkinElmer Life and Analytical Sciences, Boston, MA, USA). The samples were incubated for 7 min at 95°C for denaturation before cycles (95°C for 40 sec, 55°C for 40 sec, and 72°C for 40 sec). After 40 cycles, primer extension was continued for 7 min at 72°C. PCR products were then submitted to the restriction enzyme *MseI* (New England BioLabs, Beverly, MA, USA), analysed by 1% agarose gel electrophoresis, and visualized by ethidium bromide staining under UV light. The presence of the T1526C SNP that codes for the substitution of the stop codon by a Glutamine codon leaves the PCR fragment uncut by the restriction endonuclease *MseI*.

### RT-PCR

Saponin-treated, ring-stage parasites were harvested by centrifugation, and lysed in 10 pellet volumes of Trizol (Gibco) before freezing at −80°C. Total RNA was prepared from thawed samples following the manufacturer’s instructions. RNA was treated with DNAse I (Invitrogen) for 15 min at 37°C in presence of RNAseOUT™ inhibitor (Invitrogen). PF11_0512 mRNA was transcribed from 1 μg RNA into cDNA using Titan One tube RT-PCR kit (Roche) in a final volume of 50 μl of reaction buffer containing 0.2 mM dNTP each, 5 mM dithiothreitol, 5 unit RNAse inhibitor, 0,4 μM primer 5′ CGCTCGAGATGAAACAACATAGTTC and primer 3′ GGCAGATCTTTTTAATAGTTCATTTATTGTG, and 1 μl of enzyme mix (AMV reverse transcriptase, Taq DNA polymerase and Tgo DNA polymerase). After 30 min incubation at 48°C, the reaction was terminated at 94°C for 2 min and immediately processed for PCR amplification. Thirty-five temperature cycles were conducted as follows: denaturation at 94°C for 30 sec, annealing at 52°C for 30 sec, and extension at 68°C for 2 min 50 sec with a 3-min final time increment.

#### Cloning and sequencing of *resa2* cDNA

*Resa2* cDNA product was purified and cloned into ZERO Blunt TOPO vector (Invitrogen®) following the manufacturer’s recommendations and sequenced using MWG Eurofins company services with Sp6 and T7 primers or *resa2* specific primers.

### *Data analysis*

Data from the two patients groups of Benin and Senegal were combined to assess the association between mutant allele and severity of malaria. As some isolates harboured both wild and mutated parasites and because the proportion of each genotype was unknown, two different analyses were performed: in the first one, mixed isolates were merged with mutant isolates and compared with wild isolates; in the second analysis, mixed isolates were excluded from the analysis and mutant isolates were compared with wild isolates. Because the two groups differed in terms of seasonality of malaria transmission and in terms of age, the odds ratio of mutant allele were adjusted for age and country. Groups were compared using the Wilcoxon ranksum test for continuous variables and the Fisher’s exact test for categorical variables. Then a logistic regression was conducted to identify factors associated with mutant alleles, and to estimate odds ratios and 95% confidence intervals (CI) for the association between exposure variables and mutant alleles. Statistical analysis was performed using Stata 10 (Stata Corporation, College Station, TX, USA).

## Results

### Gene structure

Previous predictions based on alignment with *resa1* gene generated a gene model with a single intron and an internal TAA stop codon in *resa2* exon 2 [16]. Partial sequencing of the *resa2* mRNA expressed *in vivo* by parasites collected from an infected *Saimiri* monkey was consistent with this model [19]. However, the actual bio-informatic prediction in PlasmodB (PlasmodB 8.1) indicates the presence of two additional introns, which once spliced out, remove the stop codon in the *resa2* mRNA.

To clarify the gene structure, amplification of *resa2* cDNA by RT-PCR was performed using *P. falciparum* RNA purified from a patient isolate and the K1 culture adapted strain. Sequencing analysis of cloned RT-PCR fragment product showed that a single intervening sequence is spliced out in the final mRNA. A very minor band, about 100 bp smaller, was also visible following RT-PCR, and may correspond to the PlasmodB predicted cDNA. However cloning failure of this minor product excluded assessment of this prediction. A unique ORF with the described premature translation stop was found in the patient isolate [Genbank access number: JN183870], while the K1 strain harboured the T1526C mutation [Genbank access number: JN183869]. Thus, *resa2* behaved as a transcribed pseudogene in some strains harbouring the T1526 allele, and as a normal gene in those possessing a 1526C allele, which codes for a full length coding sequence.

PF11_0512 sequence available in both PlasmodB and GenedB database also differs from the original sequence by a frameshift in the coding sequence, an event not detected in both K1 and SAMA cloned cDNA sequence reported here. This frameshift results from different A residues in a polyA stretch, which was found to be associated to frequent polymerase slippage as evidenced by sequencing of many uncloned or cloned PCR products encompassing this region. Interestingly, Blast analysis against *P. falciparum* sequences available at Broad Institute or Sanger Institute indicated that number of A residues in this genomic region may differ; some strains, such as Santa Lucia strain or Ghanaian isolate shared a similar number of A with SAMA and K1 parasites.

### T1526C mutation in field isolates

The proportion of isolates harbouring wild, mutant and mixed alleles did not significantly differ between groups from Senegal and Benin (p = 0.4). The proportion of SM and UM cases did not differ either in these groups (p = 0.2). However age ranges were significantly different between the groups (p < 0.0001) as more adults were included in Senegal.

### Analysis including mixed isolates

The amplification of the *resa2* region encompassing the T1526C mutation was obtained in 39 SM isolates and 21 UM isolates from Senegal ( Additional file 1 and Additional file 2). RFLP analysis using *MseI* enzyme was performed as the restriction site is affected by the T1526C mutation. The T1526C mutation was found in six of the SM isolates (15.4%). In UM isolates, only one sample harboured the mutant allele (4.8%) in association with the wild allele. The mutant allele was not found more frequently in SM cases than in UM cases (p = 0.2). The presence of the mutant allele was not associated either with parasitaemia (p = 0.3).

In the group of Beninese children, *resa2* PCR amplification was successful for all isolates. Sixteen isolates (15.8%) harboured the T1526C allele, 11 among the severe cases and five among the UM cases. Six isolates (three corresponding to SM and three to UM) harboured both wild and mutant alleles. The mutant allele was not found more frequently in SM cases than in UM cases (p = 0.2) (Table 1). There was a trend towards the association of the presence of the mutant allele and parasitaemia > 4% (p = 0.06).

**Table 1:** **Factors associated with the presence of the*resa2*T1526C allele in*P. falciparum*infected Beninese patients. Analysis with mixed isolates**

	**T1526C SNP**	**Wild allele**	**p valu**e
Infected patients n (%)	16 (16)	85 (84)	
Variables			
**Sex**			
Male	8 (50)	53 (62)	0.3
Female	8 (50)	32 (38)	
**Age** (months)			
Median (min-max)	39 (7–120)	48 (4–114)	
In quartile			
<=24 months	6 (38)	24 (28)	
24.1-48	4 (25)	22 (26)	0.9
48.1-84	3 (19)	17 (20)	
>84	3 (19)	22 (26)	
**Malaria**			
Severe	11 (69)	43 (51)	0.2
Uncomplicated	5 (31)	42 (49)	
**Parasitaemia (%)**			
Median (min-max)	7 (0.2-36)	3 (0.01-65)	0.2
By category			
>4%	10 (63)	32 (38)	0.06
<=4%	6 (37)	53 (62)	
**Malaria prophylaxis**			
Yes	5 (42)	30 (41)	0.9
No	7 (58)	44 (59)	
**Splenomegalia**			
Yes	5 (31)	27 (32)	0.9
No	11 (69)	58 (78)	

When data from Senegal and Benin were pooled, severity of cases was not significantly associated but showed a trend towards association with the *resa2* T1526C mutation (p = 0.09).

### Analysis excluding mixed isolates

In the group of Senegalese patients, there was a trend towards the association of the presence of the mutant allele and severity of malaria (p = 0.06). The presence of the mutant allele was not associated with parasitaemia (p = 0.3).

In the group of Beninese children, there was a trend towards the association of the presence of the mutant allele and severity of malaria (p = 0.08) (Table 2). There was a trend towards the association of the presence of the mutant allele and parasitaemia > 4% (p = 0.05). In multivariate analysis, this association became statistically significant (p = 0.03) (Table 3).

**Table 2:** **Factors associated with the presence of the*resa2*T1526C allele in*P. falciparum*infected Beninese patients. Analysis without mixed isolates**

	**T1526C SNP**	**Wild allele**	**p value**
Infected patients n (%)	10 (10,5)	85 (89,5)	
Variables			
**Sex**			
Male	8 (80)	53 (62)	0.3
Female	2 (20)	32 (38)	
**Age** (months)			
Median (min-max)	35 (24–108)	48 (4–114)	0.5
In quartile			
<=24 months	4 (40)	24 (28)	
24.1-48	2 (20)	22 (26)	0.6
48.1-84	3 (30)	17 (20)	
>84	1 (10)	22 (26)	
**Malaria**			
Severe	8 (80)	43 (51)	0.08
Uncomplicated	2 (20)	42 (49)	
**Parasitaemia (%)**			
Median (min-max)	7 (0.2-36)	3 (0.01-65)	0.2
By category			
>4%	7 (70)	32 (38)	0.05
<=4%	3 (30)	53 (62)	
**Malaria prophylaxis**			
Yes	3 (38)	30 (41)	0.8
No	5 (62)	44 (59)	
**Splenomegalia**			
Yes	2 (20)	27 (32)	0.4
No	8 (80)	58 (78)	

**Table 3:** **Multivariate analysis of factors associated with the*resa2*T1526C allele in*P. falciparum*infected Beninese patients (n = 95)**

**Variables**	**Odds ratio**	**95% confidence interval**	**p value**
**Parasitaemia**			
<=4%	1		
>4%	5.7	1.2-26.9	0.03
**Sex**			
Female	1		
Male	8.3	0.8-83.7	0.07

When data from Senegal and Benin were pooled, severity of cases was significantly associated with the *resa2* T1526C mutation, Odds Ratio = 5.7; Confidence Interval 95% : [1.26-26.1](p = 0.008). The association between the presence of mutant allele and severity of malaria remained significant when age and origin of isolates (Benin or Senegal) were controlled for in a bivariate analysis.

### Association of T1526C mutation and *in vitro* growth rate

Multiplication rates of *P. falciparum* parasites isolated from SM or UM patients in Benin patients were compared in a short-term, 72-hour *in vitro* culture growth assay (Table 4). After 72 hours *in vitro* culture, parasite multiplication rate over 1.5 was obtained for 19 SM isolates (37%) and 7 UM isolates (16%). Isolates showing higher multiplication rates were more frequently associated with SM (odds ratio 5.9, IC95% 1.6-21.6) (Table 4). Five out of the eight SM isolates (62.5%) carrying the *resa2* T1526C allele have multiplication rates ranging from 1.5 to 4.3. Two out of three SM parasites with the T1526 mutation that failed to grow were collected from patients who had taken anti-malarial prophylaxis. Growth culture failed for all mixed isolates. All UM parasites with parasite multiplication rate over 1.5 have the wild *resa2* allele.

**Table 4:** Association of the parasite multiplication rate (interquartiles) with severe malaria in 97 Beninese patients

	**Severe Malaria (n = 53)**	**Uncomplicated Malaria (n = 44)**	**Odds ratio severe malaria**	**95% confidence interval**	**p**
Parasite multiplication rate (PMR)					
≤0.1	13 (25)	8 (18)	3.5	0.9-13.02	
0.11-0.7	15 (28)	16 (36)	2.03	0.6-6.7	0.03
0.71-1.7	6 (12)	13 (30)	1		
>1.7	19 (35)	7 (16)	5.9	1.6-21.6	

## Discussion

As *P. falciparum* is haploid in humans, isolates containing both *resa2* T1526C mutant and wild alleles corresponded to multiclonal isolates. Multilocus genotyping of the Senegalese isolates showed quite a large genetic diversity, and importantly outlined that most patients (SM and UM) were infected by at least two distinct clones [21]. In the present study some isolates (one UM case Senegal, three SM cases and three UM cases in Benin) harboured both wild and mutant *resa2* alleles. As the proportion of each allele within those mixed isolates was unknown, it appeared difficult to correlate the severity of the clinical access with the presence of *resa2* mutant for those isolates. Indeed, the analysis including those mixed isolates did not show an association of the presence of the mutant allele and severity of malaria. Conversely, such an association was found in the analysis excluding the mixed isolates. In addition, an association of the presence of the mutant allele and parasitaemia > 4% was found in the group of Beninese children.

To gain statistical power, data from Beninese and Senegalese groups were combined to assess the association between mutant allele and severity of malaria. This association, observed for the combined series (n = 154), was not significant for each group alone probably due to a lack of power, but a trend towards this association was found for each group.

About 14% of isolates obtained from Senegal and Benin harboured mutant T1526C parasites. Sequencing of *resa2* cDNAs showed that the mutation T1526C affects an internal stop codon and restores a unique open reading frame. Thus, all mutant parasites theoretically have the ability to encode a full-length protein. RESA2 protein as RESA1 has a PEXEL motif and is member of the pHISTb (*Plasmodium* helical interspersed subtelomeric family) protein family [26] suggesting that as its sister protein RESA1, it could be secreted out of the parasite and contribute *in vivo* to remodelling the host cell and possibly the erythrocyte membrane so as to favour high-density infections.

In multivariate analysis, parasitaemia >4%, appeared as a factor associated with the presence of mutant T1526C parasites in the group of Beninese children. It should be stressed that parasitaemia >4% is by itself a criterion, including the case in the severe category of malaria attack. Thus, it is not surprising that the presence of mutant T1526C parasites was associated with both parasitaemia >4% and SM. Indeed, it has been reported that *P. falciparum* isolates from patients with severe malaria had higher multiplication rates *in vitro* than those from uncomplicated malaria [27]. A similar observation was obtained in the series from Benin using short-term, 72-hour *in vitro* culture growth assay. Moreover, five out of six parasites harbouring the T1526C mutation, and collected from patients who had not taken anti-malarial prophylaxis, were able to multiply in culture, a prevalence far above the one observed for parasites carrying the wild allele. A rapid multiplication rate leading to high parasite density before an effective protective response is attained may contribute to severity [27-29].

Most severe cases of the series (43 of 51 in Benin, 33 of 39 in Senegal) were not due to parasites harbouring the T1526C mutation. There is no simple one-to-one correlation between the clinical syndrome and the pathogenic processes involved in SM, probably reflecting the contribution of multiple host and parasite factors to malaria pathogenesis. Yet the T1526C mutation is one of the first parasite SNPs associated with SM. Further work is needed to understand how the RESA2 protein contributes to pathogenesis and whether or not it fulfils a role similar to RESA1 in providing the parasites with a better fitness and survival under febrile conditions.

## Conclusions

There is an urgent need to better understand the pathogenesis of malaria, especially its severe forms. Identification of parasite factors that contribute to increased fitness or are targeted by host defense responses may lead to new diagnostic, prognostic and therapeutic tools. Parasite virulence factors are probably intrinsic components of the process that leads to SM. However, the confounding effect of the rapidly acquired immunity to these life-threatening parasites and the potential bias due to host genetic background could hamper their detection. Other studies in various geographic settings and probably including more patients are now required to replicate these results and to answer the questions raised by these results.

## Abbreviations

SM = Severe malaria; UM = Uncomplicated malaria; PBS = Phosphate-buffered saline; RESA = Ring erythrocyte surface antigen; PCR = Polymerase chain reaction; RT-PCR = Reverse transcriptase-Polymerase chain reaction; PEXEL = Protein export element.

## Competing interest

The authors declare that they have no competing interest.

## Authors’ contributions

RD, FMN, ES, PD, OMP and SB conceived and designed the project. FMN, FV, GS, and FL performed clinical studies. RD, FMN, VA, and SB performed experiments. RD, VA, ES, FV, GS, FL and SB analysed the data. RD and SB prepared the manuscript. FMN, OMP and PD critically reviewed the manuscript. All authors read and approved the final manuscript.

## Supplementary Material

Additional file 1 **Characteristics of 39 Senegalese patients presenting with severe*P. falciparum*malaria.** Click here for file

Additional file 2 **Characteristics of 30 Senegalese patients presenting with uncomplicated*P. falciparum*malaria.** Click here for file

Additional file 3 **Characteristics of 54 Beninese patients presenting with severe*P. falciparum*malaria.** Click here for file

Additional file 4 **Characteristics of 47 Beninese patients presenting with uncomplicated*P. falciparum*malaria.** Click here for file
